# Radical Spin Polarization and Magnetosensitivity from Reversible Energy Transfer

**DOI:** 10.1021/acs.jpclett.4c00656

**Published:** 2024-04-09

**Authors:** John M. Hudson, Emrys W. Evans

**Affiliations:** ‡ Department of Chemistry, Swansea University, Swansea SA2 8PP, United Kingdom; † Centre for Integrative Semiconductor Materials, Swansea SA1 8EN, United Kingdom

## Abstract

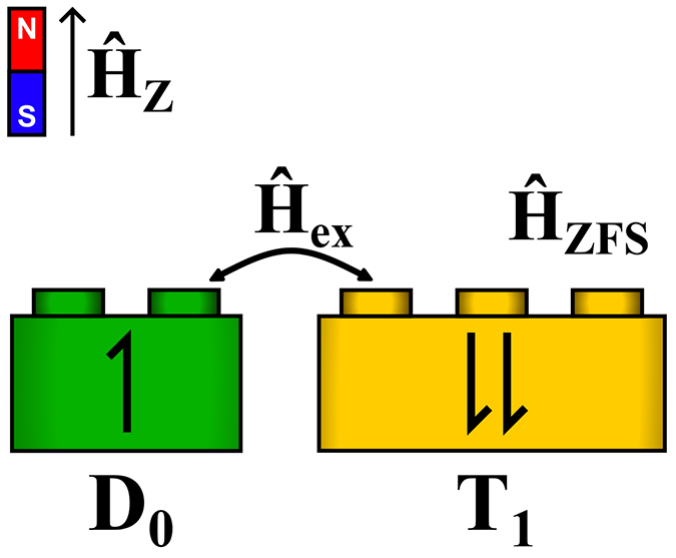

Molecular spins provide potential building units for future quantum information science and spintronic technologies. In particular, doublet (*S* = ^1^/_2_) and triplet (*S* = 1) molecular spin states have the potential for excellent optical and spin properties for these applications if useful photon-spin mechanisms at room temperature can be devised. Here we explore the potential of exploiting reversible energy transfer between triplet and doublet states to establish magnetosensitive luminescence and spin polarization. We investigate the dependence of the photon-spin mechanism on the magnitude and sign of the exchange interaction between the doublet and triplet spin components in amorphous and crystalline model systems. The design of a magnetic field inclination sensor is proposed from understanding the required “structure” (spin interactions) to “function” (magnetosensitivity).

Doublet (*S* = ^1^/_2_) and triplet (*S* = 1) spin states from molecules provide potential building units for creating designer material platforms in quantum information science (QIS) and spintronic applications.^1,2^ The challenge is to combine the potential for excellent optical and spin properties in π-conjugated organic materials and achieve useful photon-spin mechanisms at room temperature. In these systems, triplet states are derived from organic chromophore groups following photoexcitation and intersystem crossing.^3−7^ Doublet states are obtained from the unpaired electron spin in stable radicals.^8−12^ The emergence of luminescent radicals is expanding the range of spin and magnetic phenomena in doublet–triplet systems from the ground electronic state of radicals to excited states.^13^

In chromophore–radical systems, the “extra spin” of the radical can accelerate conversion between singlet (*S* = 0) and triplet chromophore states in enhanced intersystem crossing (EISC).^12,14−17^ Here it is necessary to consider the total spin of the chromophore–radical system rather than the individual moieties. A singlet chromophore (S_1_) combined with a ground state radical doublet (D_0_) forms an overall doublet state known as the sing-doublet. Triplet chromophore (T_1_) and radical doublet pairs form overall doublet and quartet (*S* = ^3^/_2_) states that may be denoted trip-doublet and trip-quartet, respectively. A spin-conserving pathway for singlet-to-triplet conversion of the chromophore moiety in EISC can proceed via sing-doublet and trip-doublet states. Efficient doublet quenching of chromophore triplet states can also become spin-allowed via trip-doublet encounter pairs, resulting in deexcitation to the sing-doublet ground state, as probed by its magnetic field dependence.^18^

In our recent work, we showed that the interplay of trip-doublet and trip-quartet states with excited D_1_ states of radicals can be exploited in demonstrations of spin initialization, manipulation, and readout by light at room temperature.^19^ The emergence of stable and luminescent radicals^20−22^ with integration into radical–triplet intermolecular^23,24^ and intramolecular systems^19^ unlocks the possibility of optical readout for studying the magnetosensitivity of radical–triplet pairs.

From previous work with nonluminescent radicals, it is established that ground state radical spin polarization may be driven by interactions with unpolarized triplet states via the Radical–Triplet(/Quartet) Pair Mechanism (RTPM/RQPM) in which radical and triplet components undergo diffusive translational motion^25,26^ and in terms of the Reversed Quartet Mechanism (RQM)^27,28^ in systems in which the components are fixed in space. In general, these mechanisms are initiated for strongly coupled radical–triplet pairs that form the trip-doublet (*M*_S_ = ±^1^/_2_, |D_±1/2_⟩) and trip-quartet (*M*_S_ = ±^3^/_2_ or ±^1^/_2_, |Q_±3/2_⟩ or |Q_±1/2_⟩, respectively). A rapid, irreversible, and spin-selective process takes place such that the trip-doublet eigenstates of the radical–triplet pair are selectively populated or depopulated. Spin polarization of the |D_+1/2_⟩ and |D_–1/2_⟩ states is generated by asymmetrical redistribution of populations to and from the trip-quartet manifold.

Here we consider radical–triplet pairs involving reversible energy transfer with excited states of luminescent radicals (D_1_ + S_0_ ⇌ D_0_ + T_1_). We explore new potential mechanisms for generating spin polarization and magnetic field inclination sensing. We investigate how magnetic interactions of the radical–triplet pair can lead to magnetic field effects with optical readout through modulating the “spin-allowed” character of energy transfer.

Starting from the framework of the Merrifield model for radical–triplet pairs,^29^ we apply a kinetic scheme in which energy transfer takes place from an excited radical doublet, D_1_, to the triplet, T_1_, via the eigenstates of the radical–triplet pair, P_*j*_:

In Scheme 1, D_1_ is generated with unpolarized light at a rate *G* (such that both doublet states are generated equally at a rate *G*/2) and can subsequently decay with a radiative rate γ_r_. Both radical doublet excited states  can undergo energy transfer to form one of the radical–triplet pair eigenstates P_*j*_ with rate constant , where  represents the overlap between the |D_±1/2_⟩ sing-doublet initial states and radical–triplet pair eigenstate P_*j*_: . Radical–triplet pair eigenstates P_*j*_ can undergo reverse transfer with rate constant  to re-form  or dissociate into separate D_0_ and T_1_ states with rate constant γ_D_. Energy transfer rates for γ_+_, γ_–_, and γ_D_ are assumed from previous studies of EISC^5,30^ and triplet–triplet^31^ systems to range from 100 ps to 10 ns (section 1 of the Supporting Information). To focus our evaluation of the spin effects from doublet–triplet energy transfer, spin relaxation is ignored, the reversibility of energy transfer is set between D_1_ + S_0_ and the pair states, and coherence effects between P_*j*_ states are ignored.

**Scheme 1:**
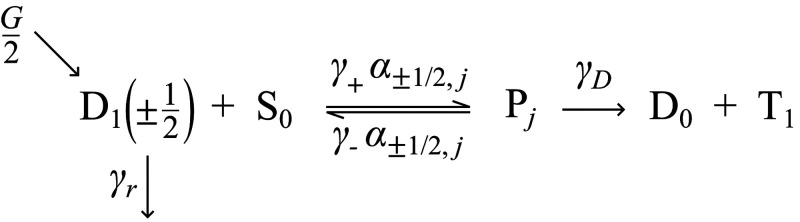
Kinetic Mechanism for Magnetosensitivity in Luminescent Radical–Triplet Systems

Radical–triplet pair eigenstates P_*j*_ are determined by the spin Hamiltonian *Ĥ*_DT_:
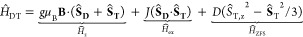
1where *Ĥ*_z_ is the Zeeman interaction for both the triplet and the radical doublet from an external magnetic field vector **B** with magnitude B, **Ŝ**_**D**_ and **Ŝ**_**T**_ are the spin operators for the doublet and triplet, respectively, μ_B_ is the Bohr magneton, and *g* is the Landé *g*-factor. *Ĥ*_ex_ is the radical–triplet exchange interaction with coupling parameter *J*, and *Ĥ*_ZFS_ represents the intramolecular zero-field splitting (ZFS) interaction of the triplet component with zero-field splitting parameter *D*.

The P_*j*_ eigenstate energies are expected to vary for typical experimental fields (<2 T) from micro- to millielectronvolts due to *Ĥ*_DT_. As the energetic separation of the D_1_ and P_*j*_ states is typically on the order of 10 meV (<*k*_B_*T* for reversible energy transfer), modulation of energy transfer rates due to energetics is assumed to be small, with spin interactions instead modulating energy transfer rates through controlling their “spin-allowed” character.

We note that this kinetic scheme is inspired by those employed by Merrifield and others to investigate the effects of magnetic fields on triplet–triplet annihilation and singlet fission in chromophore systems.^29,31−36^ The difference between triplet–triplet and radical–triplet systems is that the “spin-allowed” pathway is governed by singlet and doublet character, respectively.^18,37^ Analogous to triplet–triplet systems, the use of a kinetic model between incoherent radical–triplet states in predicting radical–triplet MFEs is expected to be most accurate where fast reversible energy transfer (time scales less than nanoseconds) causes the lifetime of P_*j*_ to be short compared to spin-coherence lifetimes (approximately nanosecond time scale between states with microelectronvolt separation). For cases in which spin-coherence between P_*j*_ states is significant, full evaluation of MFEs should be performed using density matrices for the radical–triplet pair.

Analytical solutions for excited radical doublet populations [D_±1/2_] in our radical–triplet pair system can be derived (section 2 of the Supporting Information) to write the total doublet photoluminescence, , where , , and ∑_*j*_ represents the summation over radical–triplet eigenstates P_*j*_:
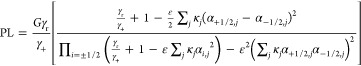
2

The doublet spin polarization in the excited state can be defined:
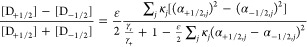
3

Spin polarization of the doublet excited state can be passed to the doublet ground state through emission. In this work, we focus on the functional spin mechanisms in the excited state.

It is apparent from the dependence of eqs 2 and 3 on  and κ_*j*_ that magnetic fields can change the doublet photoluminescence yield and excited state doublet spin polarization of D_1_. This occurs as an applied magnetic field can alter the spin character of the radical–triplet eigenstates through the Zeeman interaction.

First, we consider spin simulations of the radical–triplet system in the strongly coupled regime (|*J*| = 20|*D*|, i.e., |*J*| ≫ |*D*|). We modeled amorphous samples by setting the triplet ZFS tensor at random orientations to an applied magnetic field, where *D* = 5 μeV (typical for triplets in molecular π-conjugated systems) with a zero transverse component (*E* = 0). Figure 1 shows that for both radical–triplet systems in antiferromagnetic (*J* < 0) and ferromagnetic (*J* > 0) regimes of electron exchange, changes in doublet PL from the magnetic field effect, MFE = 100% × [PL(*B*)/PL(0) – 1], and spin polarization are observed at magnetic fields corresponding to doublet–quartet anticrossings.

**Figure 1:**
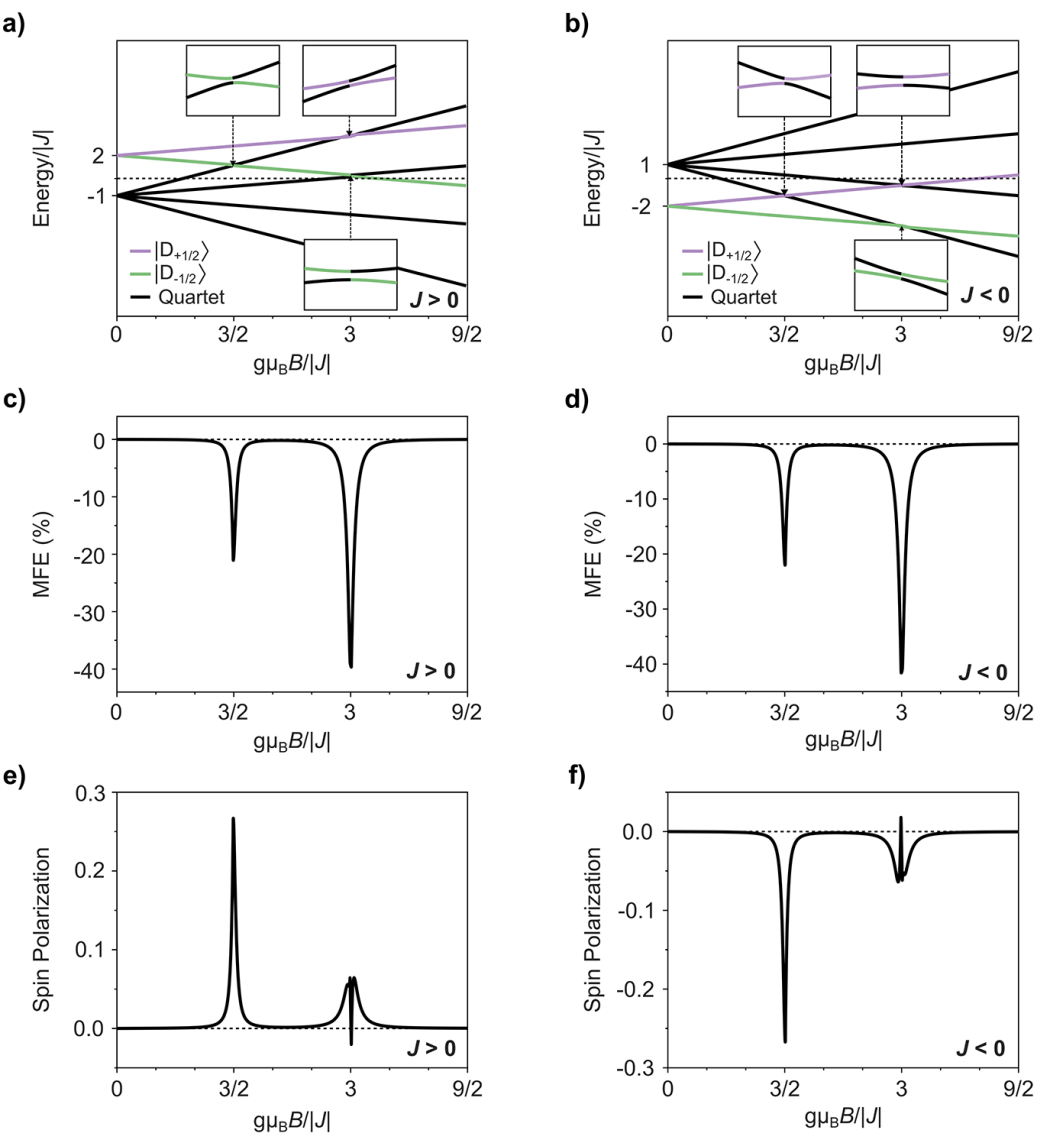
Magnetic response for strongly coupled radical–triplet pairs (|*J*| = 20|*D*|, i.e., |*J*| ≫ |*D*|) that are randomly oriented to an applied magnetic field. Zeeman splitting of radical–triplet pair states with applied magnetic field *B* for (a) ferromagnetic (*J* > 0) and (b) antiferromagnetic (*J* < 0) exchange coupling. Insets show the doublet–quartet anticrossings at triplet ZFS oriented to the applied magnetic field direction (θ = π/4). MFEs for doublet photoluminescence are shown for (c) *J* > 0 and (d) *J* < 0 radical–triplet exchange coupling. Magnetosensitivity for spin polarization from radical–triplet systems with (e) *J* > 0 and (f) *J* < 0 exchange coupling.

At zero field, the eigenstates for the strongly coupled radical–triplet pair reflect those of the radical–triplet exchange interaction, i.e., pure trip-doublet or trip-quartet spin states with energies +2*J* and −*J*, respectively. At magnetic fields (*g*μ_B_*B*) of 3|*J*|/2 and 3|*J*|, the Zeeman interaction leads to states with energy separations on the order of the ZFS parameter, |*D*|. At such fields, the ZFS interaction mixes pure spin eigenstates such that they form doublet–quartet mixtures. The hybridization of these states results in anticrossings, with the spread of trip-doublet character resulting in a decrease in the doublet photoluminescence for negative MFE, irrespective of *J* > 0 or *J* < 0.

Away from such anticrossings, application of a magnetic field alters the energy of the radical–triplet pair eigenstates but does not alter the distribution of their spin character. Consequently, no magnetic field effect (MFE) for doublet emission or spin polarization is observed outside the doublet–quartet anticrossings for strongly coupled radical–triplet pairs.

The lower-field anticrossing (*g*μ_B_*B* = 3|*J*|/2) takes place for only one of the trip-doublet states, with the asymmetrical distribution of trip-doublet character resulting in excited state doublet spin polarization. The sign of this polarization is characteristic of the sign of the exchange interaction, with *J* < 0 and *J* > 0 showing negative and positive spin polarization, respectively, as defined by eq 3.

A second field region around *g*μ_B_*B* = 3|*J*| shows spin polarization, where both trip-doublet states undergo anticrossings with trip-quartet states. In addition to a broader feature of the same spin polarization sign as at *g*μ_B_*B* = 3|*J*|/2, there is a narrow feature of inverted spin polarization. The broader feature arises for systems with ferromagnetic exchange coupling (*J* > 0) from the greater strength that the ZFS interaction mixes the |D_–1/2_⟩ and |Q_+1/2_⟩ states compared to the |D_+1/2_⟩ and |Q_+1/2_⟩ states. For a ZFS tensor oriented at angle θ to an applied magnetic field  = , while  =  (section 3 of the Supporting Information). Inversion of spin polarization arises from the doublet–quartet anticrossings at *g*μ_B_*B* = 3|*J*| being offset by the previous anticrossing at *g*μ_B_*B* = 3|*J*|/2. Radical–triplet systems with antiferromagnetic exchange coupling (*J* < 0) follow a similar trend arising from the greater strength that the ZFS interaction mixes the |D_+1/2_⟩ and |Q_–1/2_⟩ states compared to the |D_–1/2_⟩ and |Q_–3/2_⟩ states.

Strongly coupled radical–triplet systems that are crystalline (such that all triplets have a uniform orientation to the applied magnetic field) show similar MFEs at fields equivalent to 3|*J*|/2 and 3|*J*|. However, the width of magnetic response varies with triplet orientation due to different interaction strengths for spin mixing of trip-doublet and trip-quartet states by *Ĥ*_ZFS_ (section 3 of the Supporting Information).

Figure 2 shows the magnetic response for weakly coupled radical–triplet pairs (|*J*| = 0.02|*D*|, i.e., |*J*| ≪ |*D*|) that are also oriented randomly to an applied magnetic field. In contrast to the strongly coupled regime, an immediate onset of MFEs for doublet emission and spin polarization are observed.

**Figure 2:**
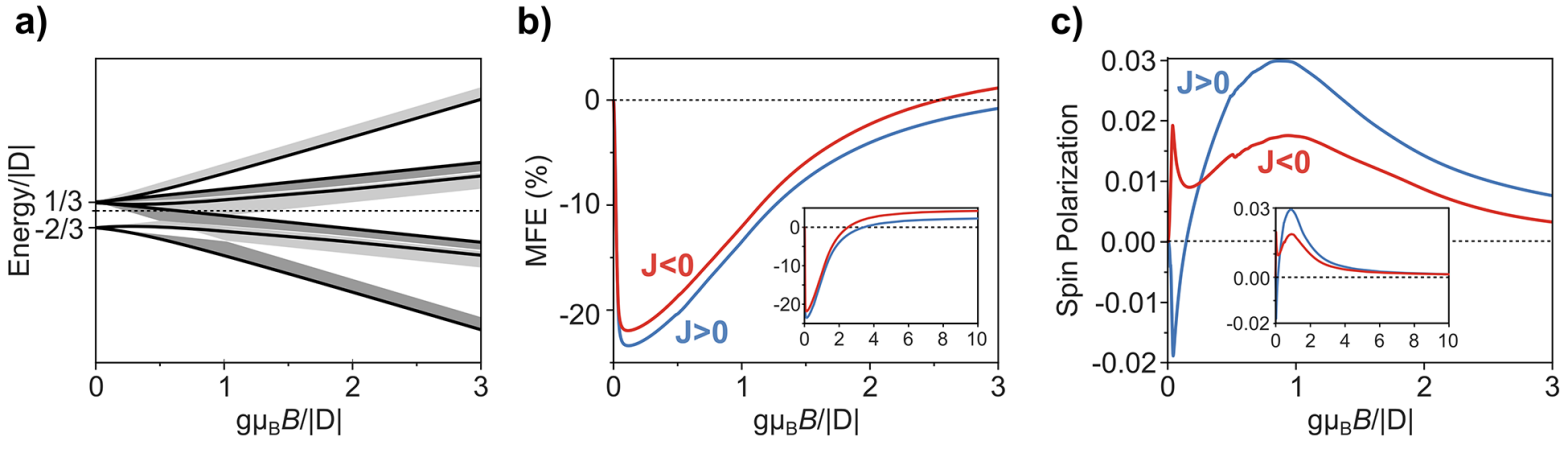
Magnetic response for weakly coupled radical–triplet pairs (|*J*| = 0.02|*D*|, i.e., |*J*| ≪ *D*|) that are randomly oriented to the applied magnetic field. (a) Zeeman splitting of radical–triplet pair states with applied magnetic field *B*. The dark line shows a radical–triplet pair for triplet ZFS tensor with θ = π/2. Gray shaded regions indicate possible energies for varying triplet orientations of an amorphous sample. (b) MFE for doublet photoluminescence from radical–triplet systems with ferromagnetic (*J* > 0; blue) and antiferromagnetic (*J* < 0; red) exchange coupling. (c) Magnetosensitivity for spin polarization from radical–triplet systems with ferromagnetic (*J* > 0; blue) and antiferromagnetic (*J* < 0; red) exchange coupling. The insets of panels b and c show the effects extending to *g*μ_B_*B* = 10|*D*|.

For weakly coupled systems, the eigenstates of the radical–triplet pair at zero magnetic field result from the triplet zero-field splitting interaction. The zero-field eigenstates are composed of linear combinations of trip-doublet and trip-quartet character. The result is a ZFS quartet and doublet with energies +*D*/3 and −2*D*/3, respectively, where two-thirds of the initial trip-doublet spin character is found within the ZFS quartet and one-third in the ZFS doublet at zero field.

The introduction of a magnetic field causes hybridization between states that are energetically separated on the order of |*J*|. At lower fields, this results in a rapid redistribution of trip-doublet character primarily within the ZFS quartet, leading to magnetic effects for the excited doublet populations that peak for fields on the order of 2|*J*|. Further changes to the distribution of trip-doublet character are seen as the magnetic field is increased above 2|*J*|, although this tends to be a limiting case for fields on the order of 2|*D*|.

For weakly coupled systems, the exact shape of the magnetic response between 0 < *g*μ_B_*B* < 2|*D*| strongly depends on the relative orientation of the triplet ZFS tensor to the applied magnetic field. This can result in magnetic responses for both the total doublet photoluminescence and spin polarization, which are dependent on triplet orientation (section 4 of the Supporting Information).

The MFEs in weakly coupled systems for both photoluminescence and spin polarization do not cancel out for disordered systems with a random triplet orientation. A characteristic decrease in photoluminescence is seen to be on the order of 2|*J*|, followed by a brightening in emission that returns toward the luminescence at zero field on the order of 2|*D*|. Photoluminescence at *B* > 2|*D*| is observed to saturate with emission greater than that at zero field.

A sharp initial feature in spin polarization is observed in weakly coupled systems: both peaking and falling on the order of 2|*J*|. This is followed by a second broader feature, peaking on the order of |*D*| before slowly decreasing to zero for systems in which the ZFS has a zero transversal component (*E* = 0). The sign of the initial sharp peak in spin polarization is determined by the sign of the exchange parameter, with *J* < 0 and *J* > 0 leading to positive and negative spin polarizations, respectively. Similarly, the second broader feature is determined by the sign of the ZFS interaction, with *D* > 0 and *D* < 0 leading to positive and negative spin polarization, respectively. Therefore, for both weakly coupled and strongly coupled chromophore–radical systems, the amorphously averaged magnetic response shows a magnetic field effect for photoluminescence that peaks on the order of |*J*| and width of |*D*|. In addition, across both systems we observe the generation of spin polarization whose sign can provide insights into the exchange and ZFS interaction for the triplet–doublet components.

We do consider that alternative interactions that result in doublet–quartet spin mixing exist apart from the ZFS interaction, including the hyperfine and a Δ*g* mechanism. The hyperfine interaction is particularly important to magnetosensitivity in radical–pair systems, with singlet–triplet spin mixing resulting in MFEs through the Radical Pair Mechanism (RPM).^38^ While the hyperfine interaction is also present in radical–triplet systems, the mixing of spin states from the ZFS interaction for molecular triplets is typically orders of magnitude greater than that from the hyperfine interaction. The effect of the hyperfine interaction on magnetosensitivity of the radical–triplet pair for this reason was not considered in this work. For radical–triplet systems, a Δ*g* mechanism can induce spin mixing, resulting in magnetic field effects. However, for purely organic materials (Δ*g* ≈ 0.001), these effects happen at fields far greater than those equivalent to |*J*| or |*D*| for strongly or weakly coupled systems, respectively (section 6 of the Supporting Information).

We note that to observe magnetosensitivity for photoluminescence and spin polarization in our kinetic scheme  must be non-zero. For large effects, systems should be engineered such that the rate of reverse triplet–doublet energy transfer is large compared to the rate of triplet dissociation. Furthermore, spin polarization will be increased for systems in which the rate of forward energy transfer (γ_+_) is maximized with respect to radiative rate (γ_r_) as seen in eq 3. Balancing the luminescent yield and dynamics that maximize the spin effects is required to optimize the optical readout for magnetosensitivity and will be investigated in our future experiments.

In the weakly coupled limit where spin mixing is driven by ZFS, the directionality of this magnetic dipolar interaction can be used as the basis of a magnetic field inclination sensor. In Figure 3, the doublet photoluminescence yield shows enhanced sensitivity, where *g*μ_B_*B* = |*D*|. The widths of the angular response are related to |*J*/*D*|. Intuitively, the magnetic field inclination sensitivity can be tailored, in principle, by engineering the spin interactions. This provides design guidelines and the basis of structure–function relationships for tuning the doublet–triplet pair through molecular “structure” modifications to target “function” in compass response.

**Figure 3:**
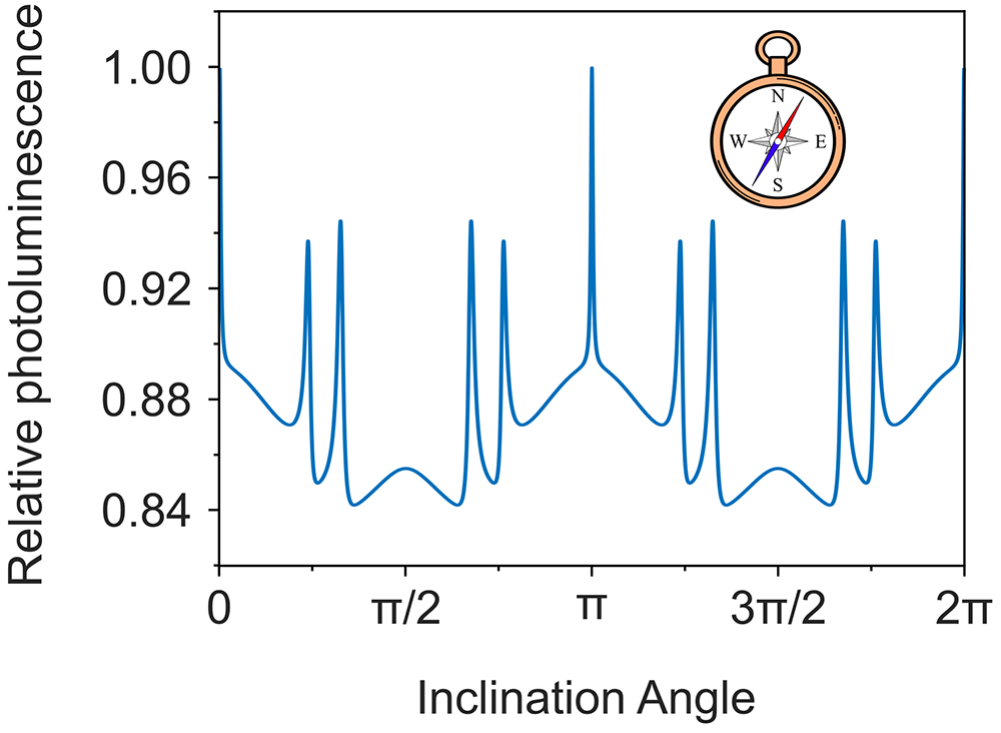
Variation in doublet photoluminescence for a weakly coupled radical–triplet pair (|*J*| = 0.02|*D*|, i.e., |*J*| ≪ |*D*|, *J* > 0) with varying inclination angle between the triplet ZFS tensor and the applied magnetic field (*g*μ_B_*B* = |*D*|). Photoluminescence values are normalized to the photoluminescence intensity for an inclination angle of 0°.

Here we examined the magnetosensitivity of energy transfer for luminescent doublet–triplet pairs from the interplay of magnetic spin interactions between molecular spins and magnetic fields. For strongly coupled doublet–triplet pairs, light readout and spin polarization initialization following reversible energy transfer reflect the size and sign of spin exchange interactions. A light-based compass sensor from tailoring spin interactions demonstrates one potential application of weakly coupled doublet–triplet pairs. Our study reveals the optical and spin properties of luminescent radical–triplet systems with potential applications in spintronic and quantum technology platforms using molecular spin materials.

## Supporting Information Available

Rate constants used for spin dynamics modeling, analytical derivations for doublet populations, magnetic response for strongly coupled (|*J*| ≫ |*D*|) systems with varying triplet orientations to an applied magnetic field, magnetic response for weakly coupled (|*J*| ≪ |*D*|) systems with varying triplet orientations to an applied magnetic field, and magnetic response for strongly and weakly coupled (|*J*| ≫ |*D*| and |*J*| ≪ |*D*|, respectively) systems with the introduction of a Δ*g* mechanism (PDF)

## References

[ref1] Wasielewski M. R.; Forbes M. D. E.; Frank N. L.; Kowalski K.; Scholes G. D.; Yuen-Zhou J.; Baldo M. A.; Freedman D. E.; Goldsmith R. H.; Goodson T.; Kirk M. L.; McCusker J. K.; Ogilvie J. P.; Shultz D. A.; Stoll S.; Whaley K. B. Exploiting Chemistry and Molecular Systems for Quantum Information Science. Nat. Rev. Chem. 2020, 4 (9), 490–504. 10.1038/s41570-020-0200-5. 37127960

[ref2] Quintes T.; Mayländer M.; Richert S. Properties and Applications of Photoexcited Chromophore-Radical Systems. Nat. Rev. Chem. 2023, 7 (2), 75–90. 10.1038/s41570-022-00453-y. 37117913

[ref3] Giacobbe E. M.; Mi Q.; Colvin M. T.; Cohen B.; Ramanan C.; Scott A. M.; Yeganeh S.; Marks T. J.; Ratner M. A.; Wasielewski M. R. Ultrafast Intersystem Crossing and Spin Dynamics of Photoexcited Perylene-3,4:9,10-Bis(Dicarboximide) Covalently Linked to a Nitroxide Radical at Fixed Distances. J. Am. Chem. Soc. 2009, 131 (10), 3700–3712. 10.1021/ja808924f. 19231866

[ref4] Colvin M. T.; Giacobbe E. M.; Cohen B.; Miura T.; Scott A. M.; Wasielewski M. R. Competitive Electron Transfer and Enhanced Intersystem Crossing in Photoexcited Covalent TEMPO–Perylene-3,4:9,10-Bis(Dicarboximide) Dyads: Unusual Spin Polarization Resulting from the Radical–Triplet Interaction. J. Phys. Chem. A 2010, 114 (4), 1741–1748. 10.1021/jp909212c. 20055506

[ref5] Avalos C. E.; Richert S.; Socie E.; Karthikeyan G.; Casano G.; Stevanato G.; Kubicki D. J.; Moser J. E.; Timmel C. R.; Lelli M.; Rossini A. J.; Ouari O.; Emsley L. Enhanced Intersystem Crossing and Transient Electron Spin Polarization in a Photoexcited Pentacene–Trityl Radical. J. Phys. Chem. A 2020, 124 (29), 6068–6075. 10.1021/acs.jpca.0c03498. 32585095

[ref6] Kirk M. L.; Shultz D. A.; Hewitt P.; Chen J.; van der Est A. Excited State Magneto-Structural Correlations Related to Photoinduced Electron Spin Polarization. J. Am. Chem. Soc. 2022, 144 (28), 12781–12788. 10.1021/jacs.2c03490. 35802385

[ref7] Kandrashkin Y.; van der Est A. Electron Spin Polarization of the Excited Quartet State of Strongly Coupled Triplet–Doublet Spin Systems. J. Chem. Phys. 2004, 120 (10), 4790–4799. 10.1063/1.1645773. 15267339

[ref8] Ito A.; Shimizu A.; Kishida N.; Kawanaka Y.; Kosumi D.; Hashimoto H.; Teki Y. Excited-State Dynamics of Pentacene Derivatives with Stable Radical Substituents. Angew. Chem., Int. Ed. 2014, 53 (26), 6715–6719. 10.1002/anie.201310291. 24788384

[ref9] Kandrashkin Y. E.; van der Est A. The Triplet Mechanism of Electron Spin Polarization in Moderately Coupled Triplet-Doublet Rigid Complexes as a Source of the Enhanced + 1/2 ↔ – 1/2 Transitions. J. Chem. Phys. 2019, 151 (18), 184301 10.1063/1.5127762. 31731838

[ref10] Mayländer M.; Chen S.; Lorenzo E. R.; Wasielewski M. R.; Richert S. Exploring Photogenerated Molecular Quartet States as Spin Qubits and Qudits. J. Am. Chem. Soc. 2021, 143 (18), 7050–7058. 10.1021/jacs.1c01620. 33929834

[ref11] Colvin M. T.; Smeigh A. L.; Giacobbe E. M.; Conron S. M. M.; Ricks A. B.; Wasielewski M. R. Ultrafast Intersystem Crossing and Spin Dynamics of Zinc Meso-Tetraphenylporphyrin Covalently Bound to Stable Radicals. J. Phys. Chem. A 2011, 115 (26), 7538–7549. 10.1021/jp2021006. 21630656

[ref12] Wang Z.; Zhao J.; Barbon A.; Toffoletti A.; Liu Y.; An Y.; Xu L.; Karatay A.; Yaglioglu H. G.; Yildiz E. A.; Hayvali M. Radical-Enhanced Intersystem Crossing in New Bodipy Derivatives and Application for Efficient Triplet-Triplet Annihilation Upconversion. J. Am. Chem. Soc. 2017, 139 (23), 7831–7842. 10.1021/jacs.7b02063. 28524657

[ref13] Teki Y. Excited-State Dynamics of Non-Luminescent and Luminescent π-Radicals. Chem. - Eur. J. 2020, 26 (5), 980–996. 10.1002/chem.201903444. 31479154

[ref14] Gijzeman O. L. J.; Kaufman F.; Porter G. Quenching of Aromatic Triplet States in Solution by Nitric Oxide and Other Free Radicals. J. Chem. Soc., Faraday Trans. 2 1973, 69, 727 10.1039/f29736900727.

[ref15] Mayländer M.; Nolden O.; Franz M.; Chen S.; Bancroft L.; Qiu Y.; Wasielewski M. R.; Gilch P.; Richert S. Accessing the Triplet State of Perylenediimide by Radical-Enhanced Intersystem Crossing. Chem. Sci. 2022, 13 (22), 6732–6743. 10.1039/D2SC01899C. 35756510 PMC9172295

[ref16] Kobori Y.; Kawai A.; Obi K. Direct Observation of CIDEP Generated through Enhanced Intersystem Crossing. J. Phys. Chem. 1994, 98 (26), 6425–6429. 10.1021/j100077a001.

[ref17] Zhang X.; Sukhanov A. A.; Yildiz E. A.; Kandrashkin Y. E.; Zhao J.; Yaglioglu H. G.; Voronkova V. K. Radical-Enhanced Intersystem Crossing in a Bay-Substituted Perylene Bisimide-TEMPO Dyad and the Electron Spin Polarization Dynamics upon Photoexcitation*. ChemPhysChem 2021, 22 (1), 55–68. 10.1002/cphc.202000861. 33197104

[ref18] Ern V.; Merrifield R. E. Magnetic Field Effect on Triplet Exciton Quenching in Organic Crystals. Phys. Rev. Lett. 1968, 21 (9), 609–611. 10.1103/PhysRevLett.21.609.

[ref19] Gorgon S.; Lv K.; Grüne J.; Drummond B. H.; Myers W. K.; Londi G.; Ricci G.; Valverde D.; Tonnelé C.; Murto P.; Romanov A. S.; Casanova D.; Dyakonov V.; Sperlich A.; Beljonne D.; Olivier Y.; Li F.; Friend R. H.; Evans E. W. Reversible Spin-Optical Interface in Luminescent Organic Radicals. Nature 2023, 620 (7974), 538–544. 10.1038/s41586-023-06222-1. 37587296 PMC10432275

[ref20] Gamero V.; Velasco D.; Latorre S.; López-Calahorra F.; Brillas E.; Juliá L. 4-(N-Carbazolyl)-2,6-Dichlorophenyl]Bis(2,4,6-Trichlorophenyl)Methyl Radical an Efficient Red Light-Emitting Paramagnetic Molecule. Tetrahedron Lett. 2006, 47 (14), 2305–2309. 10.1016/j.tetlet.2006.02.022.

[ref21] Peng Q.; Obolda A.; Zhang M.; Li F. Organic Light-Emitting Diodes Using a Neutral π Radical as Emitter: The Emission from a Doublet. Angewandte Chemie - International Edition 2015, 54 (24), 7091–7095. 10.1002/anie.201500242. 25916621

[ref22] Ai X.; Evans E. W.; Dong S.; Gillett A. J.; Guo H.; Chen Y.; Hele T. J. H.; Friend R. H.; Li F. Efficient Radical-Based Light-Emitting Diodes with Doublet Emission. Nature 2018, 563 (7732), 536–540. 10.1038/s41586-018-0695-9. 30464267

[ref23] Han J.; Jiang Y.; Obolda A.; Duan P.; Li F.; Liu M. Doublet-Triplet Energy Transfer-Dominated Photon Upconversion. J. Phys. Chem. Lett. 2017, 8 (23), 5865–5870. 10.1021/acs.jpclett.7b02677. 29144138

[ref24] Wei Y.; An K.; Xu X.; Ye Z.; Yin X.; Cao X.; Yang C. Π-Radical Photosensitizer for Highly Efficient and Stable Near-Infrared Photon Upconversion. Adv. Opt Mater. 2024, 12, 2301134 10.1002/adom.202301134.

[ref25] Kawai A.; Obi K. First Observation of a Radical-Triplet Pair Mechanism (RTPM) with Doublet Precursor. J. Phys. Chem. 1992, 96 (1), 52–56. 10.1021/j100180a014.

[ref26] Blättler C.; Jent F.; Paul H. A Novel Radical-Triplet Pair Mechanism for Chemically Induced Electron Polarization (CIDEP) of Free Radicals in Solution. Chem. Phys. Lett. 1990, 166 (4), 375–380. 10.1016/0009-2614(90)85046-F.

[ref27] Rozenshtein V.; Berg A.; Stavitski E.; Levanon H.; Franco L.; Corvaja C. Electron Spin Polarization of Functionalized Fullerenes. Reversed Quartet Mechanism. J. Phys. Chem. A 2005, 109 (49), 11144–11154. 10.1021/jp0540104. 16331897

[ref28] Qiu Y.; Equbal A.; Lin C.; Huang Y.; Brown P. J.; Young R. M.; Krzyaniak M. D.; Wasielewski M. R. Optical Spin Polarization of a Narrow-Linewidth Electron-Spin Qubit in a Chromophore/Stable-Radical System. Angew. Chem., Int. Ed. 2023, 62 (6), e202214668 10.1002/anie.202214668. PMC10107609 36469535

[ref29] Merrifield R. E. Magnetic Effects on Triplet Exciton Interactions. Pure Appl. Chem. 1971, 27 (3), 481–498. 10.1351/pac197127030481.

[ref30] Imran M.; Taddei M.; Sukhanov A. A.; Bussotti L.; Ni W.; Foggi P.; Gurzadyan G. G.; Zhao J.; Di Donato M.; Voronkova V. K. Radical-Enhanced Intersystem Crossing in Perylene-Oxoverdazyl Radical Dyads. ChemPhysChem 2022, 23 (8), e202100912 10.1002/cphc.202100912. 35191573

[ref31] Bossanyi D. G.; Sasaki Y.; Wang S.; Chekulaev D.; Kimizuka N.; Yanai N.; Clark J. Spin Statistics for Triplet-Triplet Annihilation Upconversion: Exchange Coupling, Intermolecular Orientation, and Reverse Intersystem Crossing. JACS Au 2021, 1 (12), 2188–2201. 10.1021/jacsau.1c00322. 34977890 PMC8715495

[ref32] Johnson R. C.; Merrifield R. E.; Avakian P.; Flippen R. B. Effects of Magnetic Fields on the Mutual Annihilation of Triplet Excitons in Molecular Crystals. Phys. Rev. Lett. 1967, 19 (6), 285–287. 10.1103/PhysRevLett.19.285.

[ref33] Merrifield R. E. Theory of Magnetic Field Effects on the Mutual Annihilation of Triplet Excitons. J. Chem. Phys. 1968, 48, 4319–4320. 10.1063/1.1669777.

[ref34] Faulkner L. R.; Bard A. J. Magnetic Field Effects on Anthracene Triplet-Triplet Annihilation in Fluid Solutions. J. Am. Chem. Soc. 1969, 91 (23), 6495–6497. 10.1021/ja01051a056.

[ref35] Bayliss S. L.; Weiss L. R.; Rao A.; Friend R. H.; Chepelianskii A. D.; Greenham N. C. Spin Signatures of Exchange-Coupled Triplet Pairs Formed by Singlet Fission. Phys. Rev. B 2016, 94 (4), 045204 10.1103/PhysRevB.94.045204.

[ref36] Bossanyi D. G.; Matthiesen M.; Wang S.; Smith J. A.; Kilbride R. C.; Shipp J. D.; Chekulaev D.; Holland E.; Anthony J. E.; Zaumseil J.; Musser A. J.; Clark J. Emissive Spin-0 Triplet-Pairs Are a Direct Product of Triplet–Triplet Annihilation in Pentacene Single Crystals and Anthradithiophene Films. Nat. Chem. 2021, 13 (2), 163–171. 10.1038/s41557-020-00593-y. 33288892

[ref37] Kucherenko M. G.; Penkov S. A. Triplet Excitons Quenching By Doublet Centers in a Nanoreactor with an External Magnetic Field. J. Appl. Spectrosc. 2021, 88 (2), 265–273. 10.1007/s10812-021-01168-6.

[ref38] Hayashi H. Introduction to Dynamic Spin Chemistry; World Scientific: Singapore, 2004; Vol. 8. 10.1142/5316

