# Supplementary material for: Radical Spin Polarization and Magnetosensitivity from Reversible Energy Transfer
Source: J Phys Chem Lett. 2024 Apr 9;15(15):4130–5. doi: 10.1021/acs.jpclett.4c00656 (PMC11033935; doi:10.1021/acs.jpclett.4c00656)
Supplement: Supplementary file 1 — jz4c00656_si_001.pdf [file jz4c00656_si_001.pdf]

Supporting Information for

# Radical Spin Polarization and Magnetosensitivity from Reversible Energy Transfer

*John M. Hudson‡†, Emrys W. Evans‡†**

‡ Department of Chemistry, Swansea University, Swansea, SA2 8PP, United Kingdom.

† Centre for Integrative Semiconductor Materials, Swansea, SA1 8EN, United Kingdom.

*Corresponding Author: emrys.evans@swansea.ac.uk

**Sections:**

1. Rate constants used for kinetic scheme
2. Analytical derivations for doublet populations
3. Magnetic response for strongly-coupled ($|J| \gg |D|$) systems with varying triplet orientation to an applied magnetic field
4. Magnetic response for weakly-coupled ($|J| \ll |D|$ ) systems with varying triplet orientation to an applied magnetic field
5. Magnetic response for strongly- and weakly-coupled ($|J| \gg |D|$ and $|J| \ll |D|$ respectively) systems with the introduction of Δg-mechanism

References

## 1. Rate constants used for kinetic scheme

| Rate | Value ($ns^{-1}$) | Source |
|---|---|---|
| $\gamma_r$ | 0.04 | $\tau$ = 25 ns assumed as typical radical excited state lifetime[1,2]. |
| $\gamma_+$ | 10 | $\tau$ = 100 ps. Utilizing the rate of radical-enhanced ISC shown in previous radical-chromophore systems[3,4]. |
| $\gamma_-$ | 1 | Chosen as $\gamma_+/10$ |
| $\gamma_D$ | 0.1 | Dissociation rate estimated for rubrene triplets from power dependence for the onset of bimolecular TTA[5]. |

## 2. Analytical derivations for doublet populations

Starting with Scheme 1:

Scheme 1. Kinetic Scheme for Magnetosensitivity in Doublet-Triplet Systems

$$\begin{matrix} \frac{G}{2}\searrow \\ \gamma_r\swarrow \end{matrix} D_1\left(\pm\frac{1}{2}\right) + S_0 \underset{\gamma_-\alpha_{\pm\frac{1}{2},j}}{\overset{\gamma_+\alpha_{\pm\frac{1}{2},j}}{\rightleftarrows}} P_j \overset{\gamma_D}{\longrightarrow} D_0 + T_1 \tag{1}$$

Expressions for the $\frac{\partial\left[D_{\pm\frac{1}{2}}\right]}{\partial t}$ and $\frac{\partial P_j}{\partial t}$ can be found, where $[D_{\pm 1/2}]$ represent the population of the excited doublet. Assuming steady state conditions for $\left[D_{+\frac{1}{2}}\right]$, $\left[D_{-\frac{1}{2}}\right]$ and $P_j$ respectively:

$$\frac{G}{2} = \gamma_r\left[D_{+\frac{1}{2}}\right] + \sum_j\left(\gamma_+\alpha_{+\frac{1}{2},j}\left[D_{+\frac{1}{2}}\right] - \gamma_-\alpha_{+\frac{1}{2},j}P_j\right) \tag{2}$$

$$\frac{G}{2} = \gamma_r\left[D_{-\frac{1}{2}}\right] + \sum_j\left(\gamma_+\alpha_{-\frac{1}{2},j}\left[D_{-\frac{1}{2}}\right] - \gamma_-\alpha_{-\frac{1}{2},j}P_j\right) \tag{3}$$

$$P_j = \frac{\gamma_+\left(\alpha_{+\frac{1}{2},j}\left[D_{+\frac{1}{2}}\right] + \alpha_{-\frac{1}{2},j}\left[D_{-\frac{1}{2}}\right]\right)}{\gamma_D + \gamma_-\left(\alpha_{+\frac{1}{2},j} + \alpha_{-\frac{1}{2},j}\right)} \tag{4}$$

Substituting $P_j$ from 4 into 2 and 3:

$$\frac{G}{2} = \gamma_r\left[D_{+\frac{1}{2}}\right] + \sum_j\left(\gamma_+\alpha_{+\frac{1}{2},j}\left[D_{+\frac{1}{2}}\right] - \varepsilon\gamma_+\alpha_{+\frac{1}{2},j}\left(\frac{\alpha_{+1/2,j}[D_{+1/2}] + \alpha_{-1/2,j}[D_{-1/2}]}{1 + \varepsilon\left(\alpha_{+\frac{1}{2},j} + \alpha_{-\frac{1}{2},j}\right)}\right)\right) \tag{5}$$

$$\frac{G}{2} = \gamma_r\left[D_{-\frac{1}{2}}\right] + \sum_j\left(\gamma_+\alpha_{-\frac{1}{2},j}\left[D_{-\frac{1}{2}}\right] - \varepsilon\gamma_+\alpha_{-\frac{1}{2},j}\left(\frac{\alpha_{+1/2,j}[D_{+1/2}] + \alpha_{-1/2,j}[D_{-1/2}]}{1 + \varepsilon\left(\alpha_{+\frac{1}{2},j} + \alpha_{-\frac{1}{2},j}\right)}\right)\right) \tag{6}$$

where $\varepsilon = \gamma_-/\gamma_D$ . Noting the separability of the terms in the summation over radical-triplet index $j$ and that $\sum_j \alpha_{\pm\frac{1}{2},j} = 1$, relations for $\left[D_{-\frac{1}{2}}\right]$ and $\left[D_{+\frac{1}{2}}\right]$ can be collected:

$$\frac{G}{2} = \gamma_+ \left( \frac{\gamma_r}{\gamma_+} + 1 - \sum_j \left( \frac{\varepsilon \left(\alpha_{+\frac{1}{2},j}\right)^2}{1 + \varepsilon\left(\alpha_{+\frac{1}{2},j} + \alpha_{-\frac{1}{2},j}\right)} \right) \right) \left[D_{+\frac{1}{2}}\right] - \varepsilon\gamma_+ \sum_j \left( \frac{\alpha_{+\frac{1}{2},j}\alpha_{-\frac{1}{2},j}}{1 + \varepsilon\left(\alpha_{+\frac{1}{2},j} + \alpha_{-\frac{1}{2},j}\right)} \right) \left[D_{-\frac{1}{2}}\right] \quad (7)$$

$$\frac{G}{2} = \gamma_+ \left( \frac{\gamma_r}{\gamma_+} + 1 - \sum_j \left( \frac{\varepsilon \left(\alpha_{-\frac{1}{2},j}\right)^2}{1 + \varepsilon\left(\alpha_{+\frac{1}{2},j} + \alpha_{-\frac{1}{2},j}\right)} \right) \right) \left[D_{-\frac{1}{2}}\right] - \varepsilon\gamma_+ \sum_j \left( \frac{\alpha_{-\frac{1}{2},j}\alpha_{+\frac{1}{2},j}}{1 + \varepsilon\left(\alpha_{+\frac{1}{2},j} + \alpha_{-\frac{1}{2},j}\right)} \right) \left[D_{+\frac{1}{2}}\right] \quad (8)$$

Using the substitution $\kappa_j = \left(1 + \varepsilon\left(\alpha_{+\frac{1}{2},j} + \alpha_{-\frac{1}{2},j}\right)\right)^{-1}$ and solving simultaneously:

$$\left[D_{\pm\frac{1}{2}}\right] = \frac{G}{2\gamma_+} \left\{ \frac{\frac{\gamma_r}{\gamma_+} + 1 + \varepsilon \sum_j \kappa_j \left( \alpha_{\pm\frac{1}{2},j}\alpha_{\mp\frac{1}{2},j} - \left(\alpha_{\mp\frac{1}{2},j}\right)^2 \right)}{\prod_{i=\pm\frac{1}{2}} \left( \frac{\gamma_r}{\gamma_+} + 1 - \varepsilon \sum_j \kappa_j\, \alpha_{i,j}{}^2 \right) - \varepsilon^2 \left( \sum_j \kappa_j \alpha_{+\frac{1}{2},j}\alpha_{-\frac{1}{2},j} \right)^2} \right\} \quad (9)$$

Using these expressions, the total doublet photoluminescence, $PL = \gamma_r \left(\left[D_{+\frac{1}{2}}\right] + \left[D_{-\frac{1}{2}}\right]\right)$, can be found:

$$PL = \frac{G\gamma_r}{\gamma_+} \left\{ \frac{\frac{\gamma_r}{\gamma_+} + 1 - \frac{\varepsilon}{2} \sum_j \kappa_j \left( \alpha_{+\frac{1}{2},j} - \alpha_{-\frac{1}{2},j} \right)^2}{\prod_{i=\pm\frac{1}{2}} \left( \frac{\gamma_r}{\gamma_+} + 1 - \varepsilon \sum_j \kappa_j \alpha_{i,j}{}^2 \right) - \varepsilon^2 \left( \sum_j \kappa_j \alpha_{+\frac{1}{2},j}\alpha_{-\frac{1}{2},j} \right)^2} \right\} \quad (10)$$

Similarly, the doublet spin polarization in the excited state can be defined:

$$\frac{\left[D_{+\frac{1}{2}}\right] - \left[D_{-\frac{1}{2}}\right]}{\left[D_{+\frac{1}{2}}\right] + \left[D_{-\frac{1}{2}}\right]} = \frac{\varepsilon}{2} \frac{\sum_j \kappa_j \left( \left(\alpha_{+\frac{1}{2},j}\right)^2 - \left(\alpha_{-\frac{1}{2},j}\right)^2 \right)}{\frac{\gamma_r}{\gamma_+} + 1 - \frac{\varepsilon}{2} \sum_j \kappa_j \left( \alpha_{+\frac{1}{2},j} - \alpha_{-\frac{1}{2},j} \right)^2} \quad (11)$$

**3. Magnetic response for strongly-coupled ($|J| \gg |D|$) radical-triplet pairs with varying triplet orientation to an applied magnetic field**

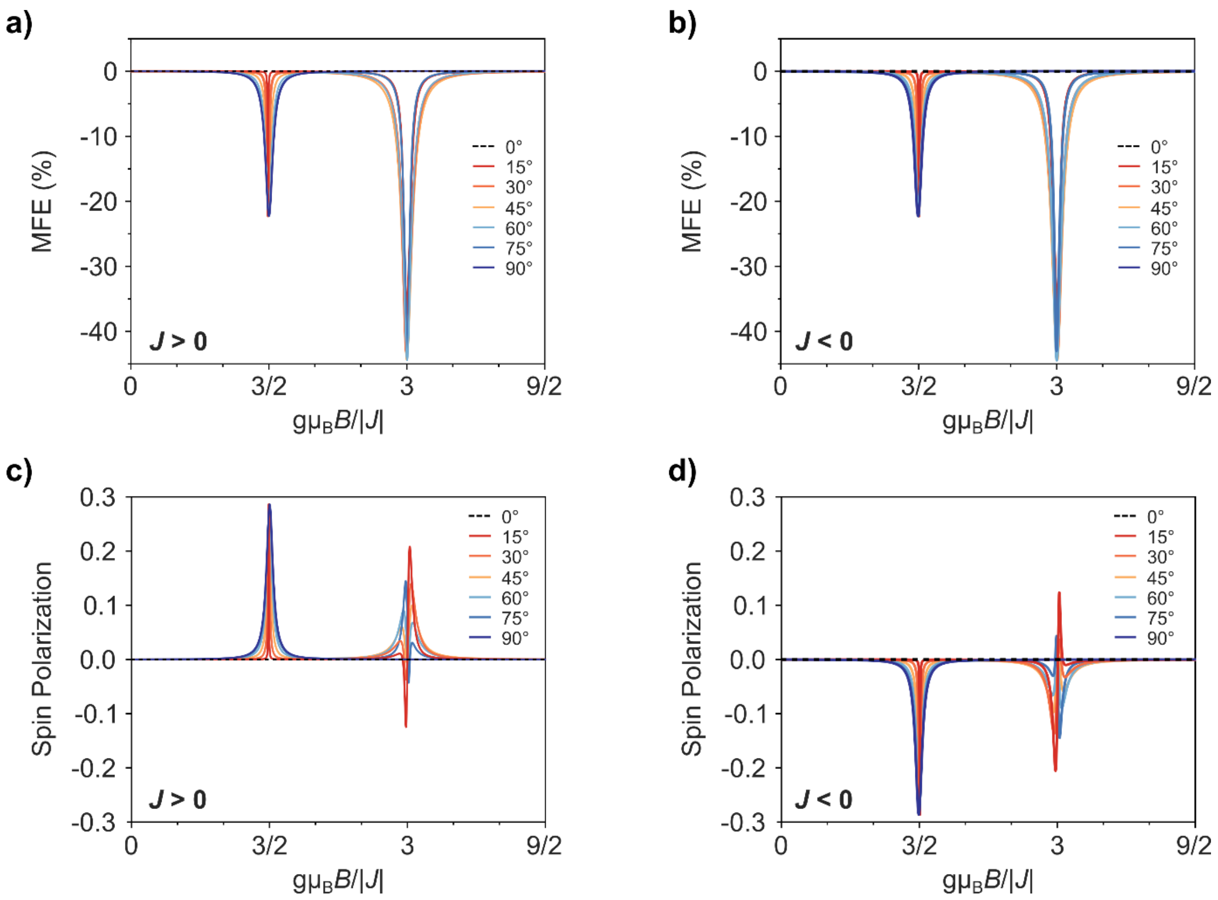


**Figure S1.** Magnetic response for a system of strongly coupled radical-triplet pairs ($|J| = 20|D|$, i.e. $|J| \gg |D|$) with all triplet states at a specified orientation to an applied magnetic field. MFEs for doublet photoluminescence are shown for (a) ferromagnetic ($J > 0$) and (b) antiferromagnetic ($J < 0$) radical-triplet exchange coupling with varying triplet ZFS tensor orientation to an applied magnetic field. MFEs for the excited doublet state spin polarization of the radical are similarly shown for (c) ferromagnetic and (b) antiferromagnetic radical-triplet exchange coupling with varying triplet ZFS orientation to an applied magnetic field.

The width of MFEs resulting from doublet-quartet anticrossings at both $g\mu_B B = 3|J|/2$ and $g\mu_B B = 3|J|$ are seen to vary with triplet orientation. For $|J| >> |D|$ we can treat the ZFS interaction ($\hat{H}_{ZFS}$) as a small perturbation to $\hat{H}^{(0)} = \hat{H}_{ex} + \hat{H}_Z$.

In strongly-coupled radical-triplet systems away from anticrossings, the radical-triplet eigenstates $|P_j\rangle$ states can be approximated with only the radical-triplet exchange and Zeeman interactions as $|P_j{}^{(0)}\rangle$ :

$$\hat{H}^{(0)}|P_j{}^{(0)}\rangle = E_j{}^0|P_j{}^{(0)}\rangle \tag{12}$$

Where $\hat{H}^{(0)} = \hat{H}_{ex} + \hat{H}_Z$ .

This results in $|P_j{}^{(0)}\rangle$ as the overall spin states for the radical-triplet system, i.e. $|D_{\pm 1/2}\rangle$, $|Q_{\pm 3/2}\rangle$, $|Q_{\pm 1/2}\rangle$.

We can use time-independent perturbation theory to estimate the radical-triplet eigenstates near to anticrossings with the addition of the ZFS interaction $|P_j{}^{(1)}\rangle$ .

To first order with non-degenerate $|P_j{}^{(0)}\rangle$ :

$$|P_j{}^{(1)}\rangle = |P_j{}^{(0)}\rangle + \sum_{j \neq k} |P_k{}^{(0)}\rangle \frac{\langle P_k{}^{(0)}|\hat{H}_{ZFS}|P_j{}^{(0)}\rangle}{E_j{}^{(0)} - E_k{}^{(0)}} \tag{13}$$

The width of MFE features for luminescence/spin polarization depends on the rate that doublet character is hybridized as an anticrossing is approached. The MFE width is affected both by the convergence rate of radical-triplet eigenstates $P_j$ at the anticrossing (i.e. relative magnetic field quantum numbers of states $k$ and $j$) and the magnitude of $\langle P_k{}^{(0)}|\hat{H}_{ZFS}|P_j{}^{(0)}\rangle$.

For a ZFS tensor with principal axis at an angle θ to the applied magnetic field, $\langle P_k{}^{(0)}|\hat{H}_{ZFS}|P_j{}^{(0)}\rangle$ can be calculated for all possible state intersections:

$$\langle Q_{+3/2}|\hat{H}_{ZFS}|D_{+1/2}\rangle = -\frac{\sqrt{6}}{12} D \sin 2\theta \tag{14}$$

$$\langle Q_{+1/2}|\hat{H}_{ZFS}|D_{+1/2}\rangle = \frac{\sqrt{2}}{12} D\,(3\cos 2\theta + 1) \tag{15}$$

$$\langle Q_{-1/2}|\hat{H}_{ZFS}|D_{+1/2}\rangle = \frac{\sqrt{2}}{4} D \sin 2\theta \tag{16}$$

$$\langle Q_{-3/2}|\hat{H}_{ZFS}|D_{+1/2}\rangle = \frac{\sqrt{6}}{6} D \sin^2 \theta \tag{17}$$

$$\langle Q_{+3/2}|\hat{H}_{ZFS}|D_{-1/2}\rangle = -\frac{\sqrt{6}}{6}D\sin^2\theta \tag{18}$$

$$\langle Q_{+1/2}|\hat{H}_{ZFS}|D_{-1/2}\rangle = \frac{\sqrt{2}}{4}D\sin 2\theta \tag{19}$$

$$\langle Q_{-1/2}|\hat{H}_{ZFS}|D_{-1/2}\rangle = -\frac{\sqrt{2}}{12}D\,(3\cos 2\theta + 1) \tag{20}$$

$$\langle Q_{-3/2}|\hat{H}_{ZFS}|D_{-1/2}\rangle = -\frac{\sqrt{6}}{12}D\sin 2\theta \tag{19}$$

For both $B = 3|J|/2$ and $B = 3J$, the width of MFE features is seen to increase with $|D|$. At $B = 3|J|/2$, the width of the MFE feature varies with $\sin^2\theta$, whilst at $B = 3|J|$ the width of both intersections varies with $\sin 2\theta$.

**4. Magnetic response for weakly-coupled (|*J*|<<|*D*|) radical-triplet pairs with varying triplet orientation to an applied magnetic field**

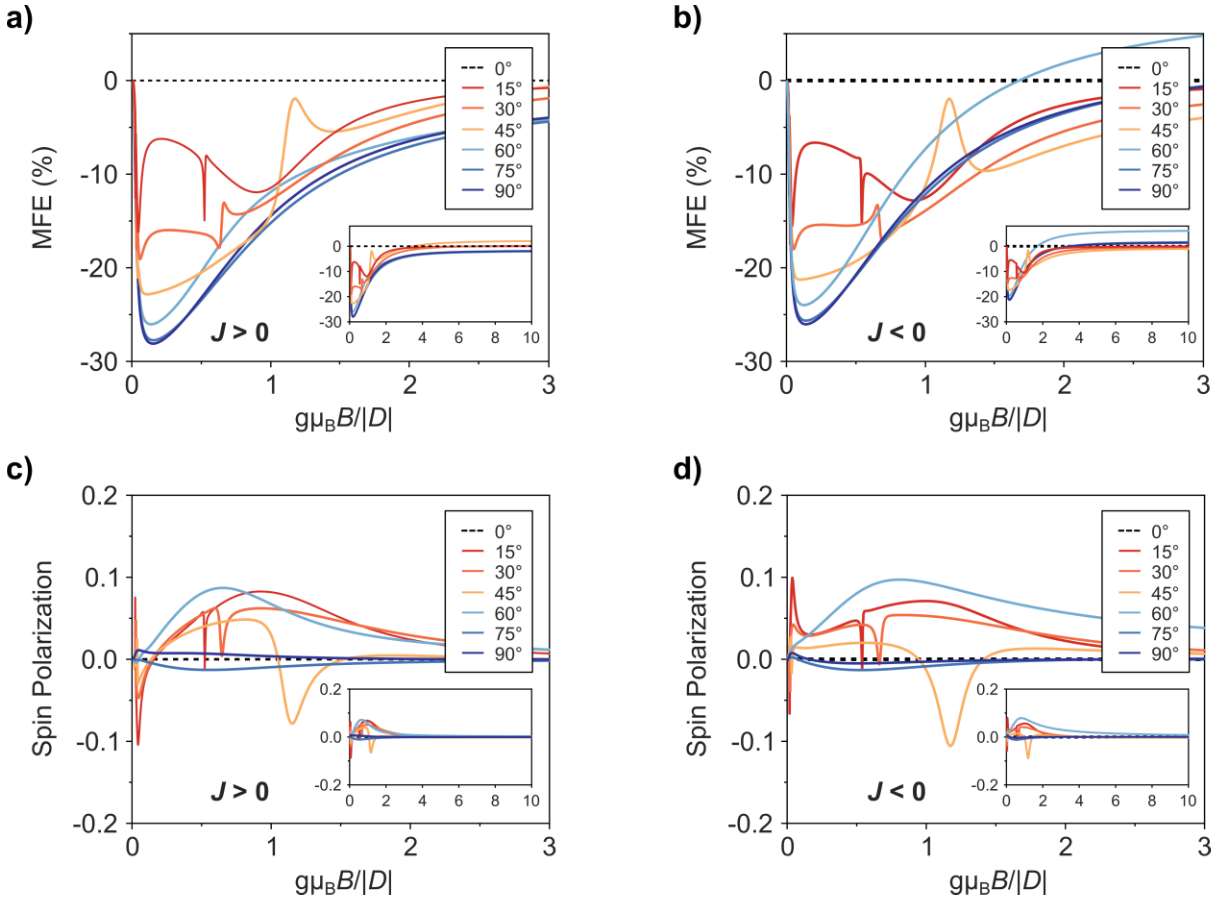


**Figure S2.** Magnetic response for a system of weakly coupled radical-triplet pairs ($|J| = 0.02|D|$, i.e. $|J| \ll |D|$) with all triplet states at a specified orientation to an applied magnetic field. MFEs for doublet photoluminescence are shown for (a) ferromagnetic ($J > 0$) and (b) antiferromagnetic ($J < 0$) radical-triplet exchange coupling with varying triplet ZFS tensor orientation to an applied magnetic field. Inset: MFEs extending up to $g\mu_B B = 10|D|$. MFEs for the excited doublet state spin polarization of the radical are similarly shown for (c) ferromagnetic and (b) antiferromagnetic radical-triplet exchange coupling with varying triplet ZFS orientation to an applied magnetic field. Inset: MFEs extending up to $g\mu_B B = 10|D|$.

**5. Magnetic response for strongly- ($|J| \gg |D|$) and weakly-coupled ($|J| \ll |D|$) radical-triplet pairs with the introduction of Δg-mechanism**

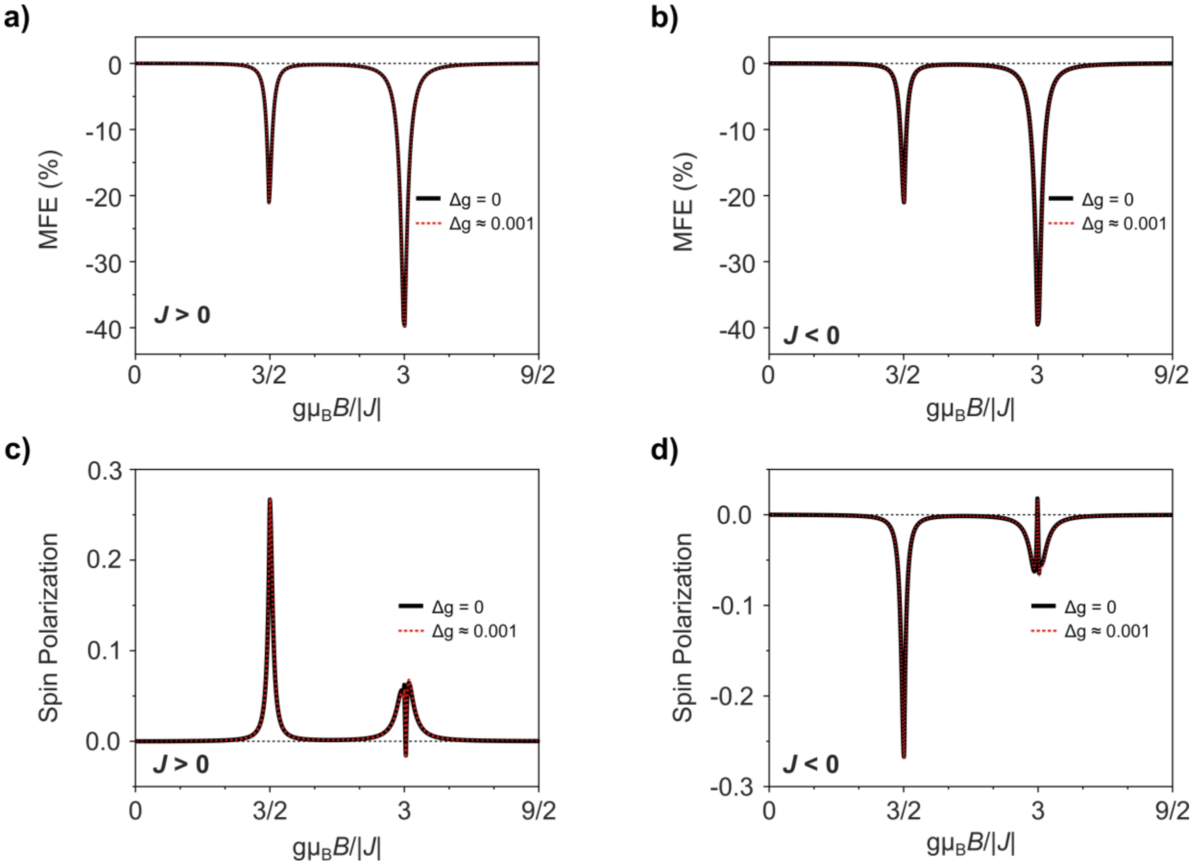


**Figure S3.** Magnetic response for a system of strongly coupled radical-triplet pairs ($|J| = 20|D|$, i.e. $|J| \gg |D|$) that are randomly oriented with respect to an applied magnetic field. Results are shown either with $g_e$ = 2.0023 for both radical and triplet (Δg = 0), or with $g_e$ = 2.0023 for radical and 2.001 for triplet (Δg ≈ 0.001). MFEs for doublet photoluminescence from radical-triplet systems with (a) ferromagnetic ($J > 0$) and (b) antiferromagnetic ($J < 0$) exchange coupling. Magnetosensitivity for spin polarization from radical-triplet systems with (c) $J > 0$ and (d) $J < 0$ exchange coupling.

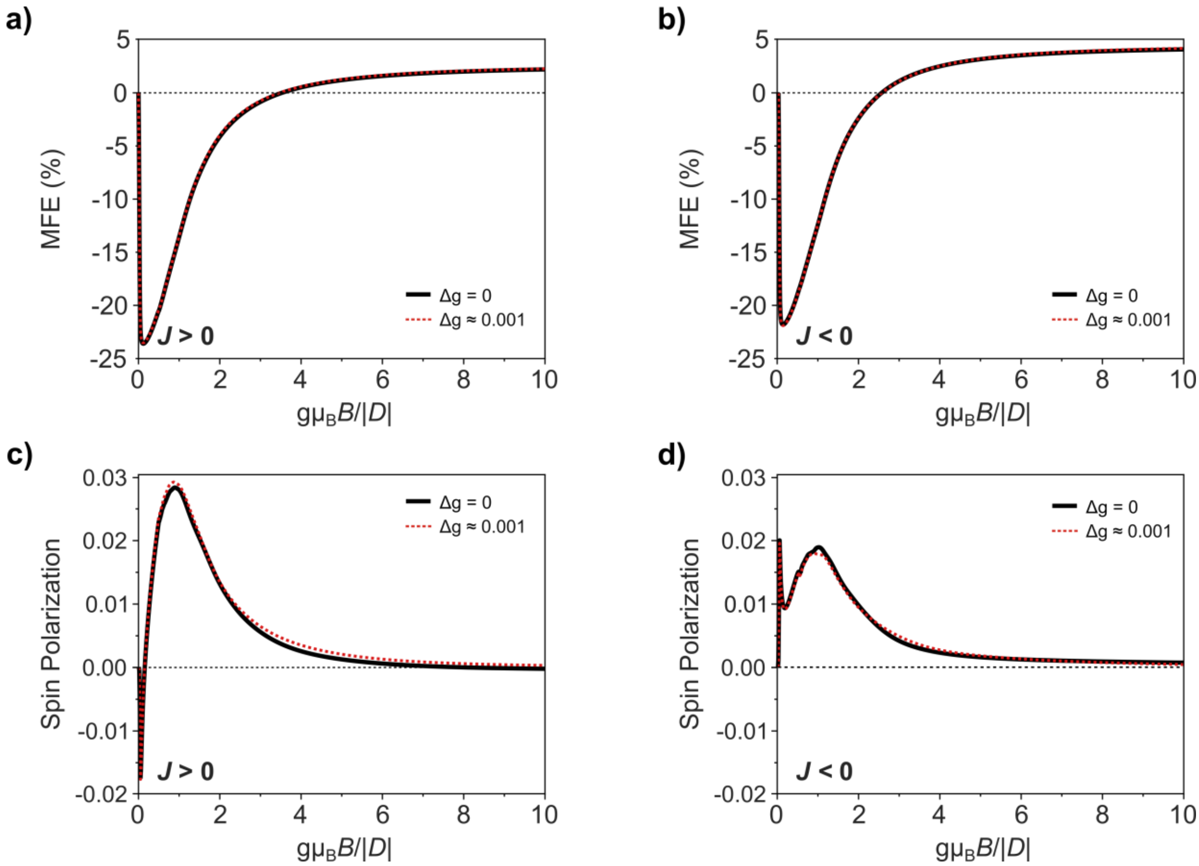


**Figure S4.** Magnetic response for a system of weakly coupled radical-triplet pairs (|$J$| = 0.02|$D$|, i.e. |$J$|>>|$D$|) that are randomly oriented with respect to an applied magnetic field. Results are shown either with $g_e$ = 2.0023 for both radical and triplet (Δg = 0), or with $g_e$ = 2.0023 for radical and 2.001 for triplet (Δg ≈ 0.001). MFEs for doublet photoluminescence from radical-triplet systems with (a) ferromagnetic ($J > 0$) and (b) antiferromagnetic ($J < 0$) exchange coupling. Magnetosensitivity for spin polarization from radical-triplet systems with (c) $J > 0$ and (d) $J < 0$ exchange coupling.

## References


(1) Ai, X.; Evans, E. W.; Dong, S.; Gillett, A. J.; Guo, H.; Chen, Y.; Hele, T. J. H.; Friend, R. H.; Li, F. Efficient Radical-Based Light-Emitting Diodes with Doublet Emission. *Nature* **2018**, *563* (7732), 536–540. https://doi.org/10.1038/s41586-018-0695-9.

(2) Hudson, J. M.; Hele, T. J. H.; Evans, E. W. Efficient Light-Emitting Diodes from Organic Radicals with Doublet Emission. *J Appl Phys* **2021**, *129* (18), 180901. https://doi.org/10.1063/5.0047636.

(3) Avalos, C. E.; Richert, S.; Socie, E.; Karthikeyan, G.; Casano, G.; Stevanato, G.; Kubicki, D. J.; Moser, J. E.; Timmel, C. R.; Lelli, M.; Rossini, A. J.; Ouari, O.; Emsley, L. Enhanced Intersystem Crossing and Transient Electron Spin Polarization in a Photoexcited Pentacene–Trityl Radical. *J Phys Chem A* **2020**, *124* (29), 6068–6075. https://doi.org/10.1021/acs.jpca.0c03498.

(4) Imran, M.; Taddei, M.; Sukhanov, A. A.; Bussotti, L.; Ni, W.; Foggi, P.; Gurzadyan, G. G.; Zhao, J.; Di Donato, M.; Voronkova, V. K. Radical-Enhanced Intersystem Crossing in Perylene-Oxoverdazyl Radical Dyads. *ChemPhysChem* **2022**, *23* (8), e202100912. https://doi.org/https://doi.org/10.1002/cphc.202100912.

(5) Bossanyi, D. G.; Sasaki, Y.; Wang, S.; Chekulaev, D.; Kimizuka, N.; Yanai, N.; Clark, J. Spin Statistics for Triplet-Triplet Annihilation Upconversion: Exchange Coupling, Intermolecular Orientation, and Reverse Intersystem Crossing. *JACS Au* **2021**, *1* (12), 2188–2201. https://doi.org/10.1021/jacsau.1c00322.
